# Reduction of sacsin levels in peripheral blood mononuclear cells as a diagnostic tool for spastic ataxia of Charlevoix–Saguenay

**DOI:** 10.1093/braincomms/fcae243

**Published:** 2024-07-18

**Authors:** Daniele De Ritis, Laura Ferrè, Jonathan De Winter, Clémence Tremblay-Desbiens, Mathieu Blais, Maria Teresa Bassi, Nicolas Dupré, Jonathan Baets, Massimo Filippi, Francesca Maltecca

**Affiliations:** Mitochondrial Dysfunctions in Neurodegeneration Unit, IRCCS Ospedale San Raffaele, 20132 Milan, Italy; Department of Neurology, IRCCS Ospedale San Raffaele, 20132 Milan, Italy; Institute Born-Bunge and Translational Neurosciences, Faculty of Medicine and Health Sciences, University of Antwerp, 2610 Antwerp, Belgium; Department of Neurology, Neuromuscular Reference Centre, Antwerp University Hospital, 2610 Antwerp, Belgium; Neuroscience Axis, CHU de Québec, Université Laval, Quebec City, QC G1V 0A6, Canada; Neuroscience Axis, CHU de Québec, Université Laval, Quebec City, QC G1V 0A6, Canada; Laboratory of Medical Genetics, Scientific Institute, IRCCS E. Medea, 23842 Bosisio Parini, Italy; Neuroscience Axis, CHU de Québec, Université Laval, Quebec City, QC G1V 0A6, Canada; Department of Medicine, Faculty of Medicine, Université Laval, Quebec City, QC G1V 0A6, Canada; Institute Born-Bunge and Translational Neurosciences, Faculty of Medicine and Health Sciences, University of Antwerp, 2610 Antwerp, Belgium; Department of Neurology, Neuromuscular Reference Centre, Antwerp University Hospital, 2610 Antwerp, Belgium; Department of Neurology, IRCCS Ospedale San Raffaele, 20132 Milan, Italy; Università Vita-Salute San Raffaele, 20132 Milan, Italy; Mitochondrial Dysfunctions in Neurodegeneration Unit, IRCCS Ospedale San Raffaele, 20132 Milan, Italy; Università Vita-Salute San Raffaele, 20132 Milan, Italy

**Keywords:** ARSACS, spastic ataxia, genetics, diagnosis, neurodegeneration

## Abstract

Autosomal recessive spastic ataxia of Charlevoix–Saguenay is a rare neurodegenerative disease caused by biallelic variants in the *SACS* gene encoding for sacsin. More than 200 pathogenic variants have been identified to date, most of which are missense. It is likely that the prevalence of autosomal recessive spastic ataxia of Charlevoix–Saguenay is underestimated due to the lack of an efficient diagnostic tool able to validate variants of uncertain significance. We have previously shown that sacsin is almost absent in fibroblasts of patients with autosomal recessive spastic ataxia of Charlevoix–Saguenay regardless of the type of *SACS* variant, because sacsin carrying missense variants is cotranslationally degraded. In this work, we aimed to establish the pathogenicity of *SACS* variants by quantifying sacsin protein in blood samples, with relevant implications for autosomal recessive spastic ataxia of Charlevoix–Saguenay diagnosis. We developed a protocol to assess sacsin protein levels by western blot using small amounts of peripheral blood mononuclear cells, which can be propagated in culture and cryopreserved. The study involves eight patients with autosomal recessive spastic ataxia of Charlevoix–Saguenay (including a novel case) carrying variants of different types and positions along the *SACS* gene and two parents who are carriers of heterozygous missense variants. We show that patients with autosomal recessive spastic ataxia of Charlevoix–Saguenay (carrying either missense or truncating variants) almost completely lacked sacsin in peripheral blood mononuclear cells. Moreover, both carriers of a *SACS* missense variant showed 50% reduction in sacsin protein levels compared to controls. We also describe a patient with uniparental isodisomy carrying a homozygous nonsense variant near the 3′ end of the *SACS* gene. This resulted in a stable sacsin protein lacking the last 202 amino acids, probably due to escape of nonsense-mediated decay of mRNA. In conclusion, we have optimized a minimally invasive diagnostic tool for autosomal recessive spastic ataxia of Charlevoix–Saguenay in blood samples based on sacsin protein level assessment. Indeed, our results provide definite evidence that sacsin carrying missense pathogenic variants undergoes cotranslational degradation. The quantitative reduction in sacsin levels in the case of missense variants of uncertain significance allows defining them as pathogenic variants, something which cannot be predicted bioinformatically with high certainty.

## Teaser

De Ritis *et al*. report a novel and minimally invasive diagnostic method for Spastic Ataxia of Charlevoix–Saguenay based on the assessment of sacsin protein levels by western blot in peripheral blood mononuclear cells. The absence of sacsin determines the pathogenicity of missense *SACS* variants, which indeed trigger sacsin cotranslational degradation.

## Graphical Abstract

**Graphical Abstract:**
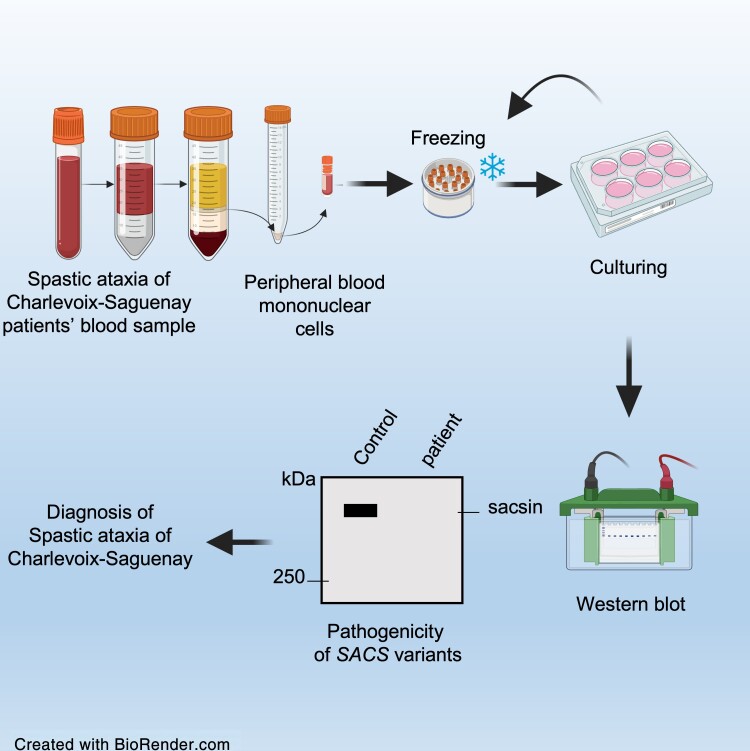

## Introduction

Autosomal recessive spastic ataxia of Charlevoix–Saguenay (ARSACS) is a rare, childhood- to adult-onset neurodegenerative disease characterized by progressive ataxia, spasticity and neuropathy.^1^ It is caused by pathogenic variants in the *SACS* gene encoding for sacsin, a 520-kDa multimodular protein whose function is still poorly understood. Sacsin is composed of an ubiquitin-like domain that binds to the proteasome,^2^ three sacsin repeating regions (SRR) having high homology with Hsp90,^3^ a *Xeroderma pigmentosum* C-binding domain,^4^ a DnaJ domain that binds Hsc70^2^ and a higher eukaryotes and prokaryotes nucleotide-binding (HEPN) domain.^5^

ARSACS is highly prevalent in the Québec French–Canadian community, due to a genetic founder effect. However, it is also present worldwide, with more than 200 pathogenic variants described,^6^ suggesting that its diagnosis and prevalence are underexplored especially in low- and middle-income countries, due to the lack of diagnostic tools.

We have previously shown that sacsin is almost absent or strikingly reduced in fibroblasts of patients with ARSACS, regardless of the nature of the *SACS* genetic variant.^7^ This applies not only to patients with ARSACS carrying truncating variants, as expected, but also to patients who are compound heterozygotes either for two diverse *SACS* pathogenic missense variants, or for a missense variant and a truncating *SACS* variant. We identified preemptive cotranslational degradation of mutant sacsin carrying pathogenic missense variants as the underlying mechanism.^7^ In this pathway, sacsin mRNA remains at a constant level and is continuously translated, but the nascent protein chain carrying pathogenic missense variant is never fully synthesized as it gets soon ubiquitinated and targeted to proteasome-mediated degradation.^7^ This mechanism seems to be universal, as the *Sacs ^R272C/R272C^* knockin mouse model presents a constant mRNA level but almost zero protein level in homozygosity and halved protein level in heterozygosity, as compared to the wild type in the cerebellum and in the brain.^8^

Therefore, we hypothesized that the measurement of sacsin protein levels in blood samples of patients with ARSACS could represent a minimally invasive tool for ARSACS diagnosis and for assessing the pathogenicity of variants of uncertain significance (VUS). Consistently, the levels of sacsin should be halved in healthy carriers of a pathogenic missense variant.

We have previously demonstrated that *SACS* gene is expressed in human blood samples from healthy controls, and in particular, sacsin mRNA and protein are mostly abundant in the peripheral blood mononuclear cells (PBMCs) fraction.^7^ Although sacsin is expressed at lower levels in PBMCs than in human fibroblasts, we hypothesized that the level is still quantifiable and allows reliable measurements, even of the 50% reduction expected in healthy carriers.

Here, we present the setup and validation of a novel protocol for the diagnosis of ARSACS in blood samples from patients which is based on the biochemical assessment of sacsin protein reduction.

## Materials and methods

### Participant consent

Eight patients (four males and four females) with the clinical and genetic diagnosis of ARSACS (Table 1), along with two parents, each related to a different one of these patients, were retrospectively recruited among the cohort of spastic–ataxic patients referring to each center: IRCCS Ospedale San Raffaele (Milan, Italy), Department of Neurology, Antwerp University Hospital (Antwerp, Belgium), and CHU de Quebec, Université Laval (Canada). All participants gave their written informed consent according to protocols in force by respective institutional human ethics review boards and Declaration of Helsinki. All reported pathogenic variants (both of patients and parents) were re-confirmed by Sanger’s sequencing.

**Table 1:** Clinical features of patients with ARSACS reported in this study

ARSACS patients	*SACS* pathogenic variants	Spasticity UL/LL	Ataxia	Dysarthria	Sensory loss	Walking difficulties/support/wheelchair	Reference (pathogenic variant description)
PN1	c.9284C > T, p.Pro3095Leu; c.3511_3512delTT, p.Leu1171Glyfs*8	−/++	+	−	−	Walking with support	Unpublished
PN2	c.11777A > G, p.Asp3926Gly; c.11265delAT, p.Ile3755fs*8	−/++	++	−	++	Walking with support	Masciullo^9^
PN3a	c.4593dupA, p.Ser1531fs*9; c.4933C > T, p.Arg1645X	−/+++	++	++	+++	Walking difficulties	Longo^7^
PN3b	c.4593dupA, p.Ser1531fs*9; c.4933C > T, p.Arg1645X	−/++	++	−/+	+++	Walking with support	Longo^7^
PN4	[c.10907G > A, p.Arg3636Gln + c.10954C > A, p.Pro3652Thr]; c.11234_11235delTT, p.Leu3745fs*1	−/++	++	++	+	Wheelchair	Baets^10^
PN5	c.4205A > T, p.Asp1402Val; c.5836T > C, p.Trp1946Arg	−/++	++	+	−	Walking with support (walker)	Thiffault^11^
PN6	c.4593dupA, p.Ser1531fs*9 homozygous	+−/+	+++	++	+++	Wheelchair	Prodi^12^
PN7	c.13132C > T, p.Arg4378X homozygous	−/+++	+++	+	+	Walking with support/wheelchair for longer distances	Anesi^13^

− absent, + − subtle, + present, ++ strongly present, +++ very strongly present. UL/LL, upper limbs/lower limbs.

All patients were evaluated in 2023 or 2024.

### Next-generation sequencing

The proband’s DNA (Patient1) was screened using a targeted next-generation sequencing (NGS) approach with a gene panel including 231 genes: all known causative genes for hereditary spastic paraplegia, the known genes for recessive ataxia and spinocerebellar ataxias (excluding those forms related to repeat expansion), the most frequently mutated genes in neuropathies and the known genes for familial ALS. Targeted NGS panel description is reported in Supplementary material.

The variants identified were verified by Sanger’s sequencing. Numbering of the pathogenic variants in *SACS* gene is based on *SACS* cDNA and protein accession numbers NM_014363.4 and NP_055178.3, respectively.

### PBMCs isolation and *in vitro* culturing

Fresh venous blood was collected in Vacutainer-EDTA tubes (BD) and PBMCs were isolated after density gradient centrifugation (Lymphoprep, STEMCELL Technologies). PBMCs were then either cryopreserved in medium with 10% dimethyl sulfoxide (DMSO) or harvested at 2 × 10^6^ cells/mL density, up to 21 days *in vitro* (DIV), with X-VIVO™ medium (Lonza) supplemented with 5% human AB male serum (ECS0219D, Euroclone), 1 mM sodium pyruvate, 2 mM l-glutamine, 100 U/mL penicillin–streptomycin, 50 UI/mL human recombinant interleukin-2 (IL-2, Sigma) and phytohaemagglutinin (PHA, Merck) at 1 µg/mL. From DIV3, cells in suspension (which are mainly lymphocytes) were washed and incubated with medium without PHA and with 100 UI/mL IL-2. Immunophenotyping at DIV6 by flow cytometry confirms high frequency of live cells comparable to fresh PBMCs (DIV0) and conserved CD3^+^ T cells percentage of lymphocytes (around 60%). Also, in CD3^+^ T cells the CD4^+^/CD8^+^ ratio was normal (around 1.1:1 at DIV6 compared to 2.3:1 at DIV0) (*data not shown*). Control PBMCs were collected as above from six different healthy volunteers (age ranging 30–40 years, both males and females).

### Western blot

Sacsin protein level was measured by western blot. Briefly, 10–30 μg of PBMCs lysed with 1% TritonX-100 was loaded on 6 or 5% SDS–PAGE. The following antibodies were used: anti-sacsin (Abcam 181190), anti-calnexin (Sigma C4731) and horseradish peroxidase-conjugated anti-rabbit IgG (GE Healthcare #GEHNA9341ML).

Densitometric analyses of western blot chemiluminescence signal were performed with ImageJ, normalizing sacsin to either calnexin or Ponceau S staining signal levels.

### Immunofluorescence on ARSACS fibroblasts

Primary fibroblasts from controls and PN6 and PN7 biopsies were cultured as we previously described.^7^ After fixation and permeabilization, the cells were incubated with anti-vimentin antibodies (Abcam Ab92547) and then Alexa Fluor-488 secondary antibodies (Thermo Fisher Scientific) and counterstained with DAPI. Images were acquired with Zeiss Axio-Observer (20× 0.5) and Volocity 6.3 software.

### Statistical analyses

One-way ANOVA with Tukey’s correction for multiple comparison was always used to compare normalized sacsin levels among controls, carriers and patients’ groups. Unpaired *t*-test was used to measure alteration of sacsin levels in PN7 compared to controls. Analyses were performed with GraphPad Prism 8.

## Results

**Figure 1:**
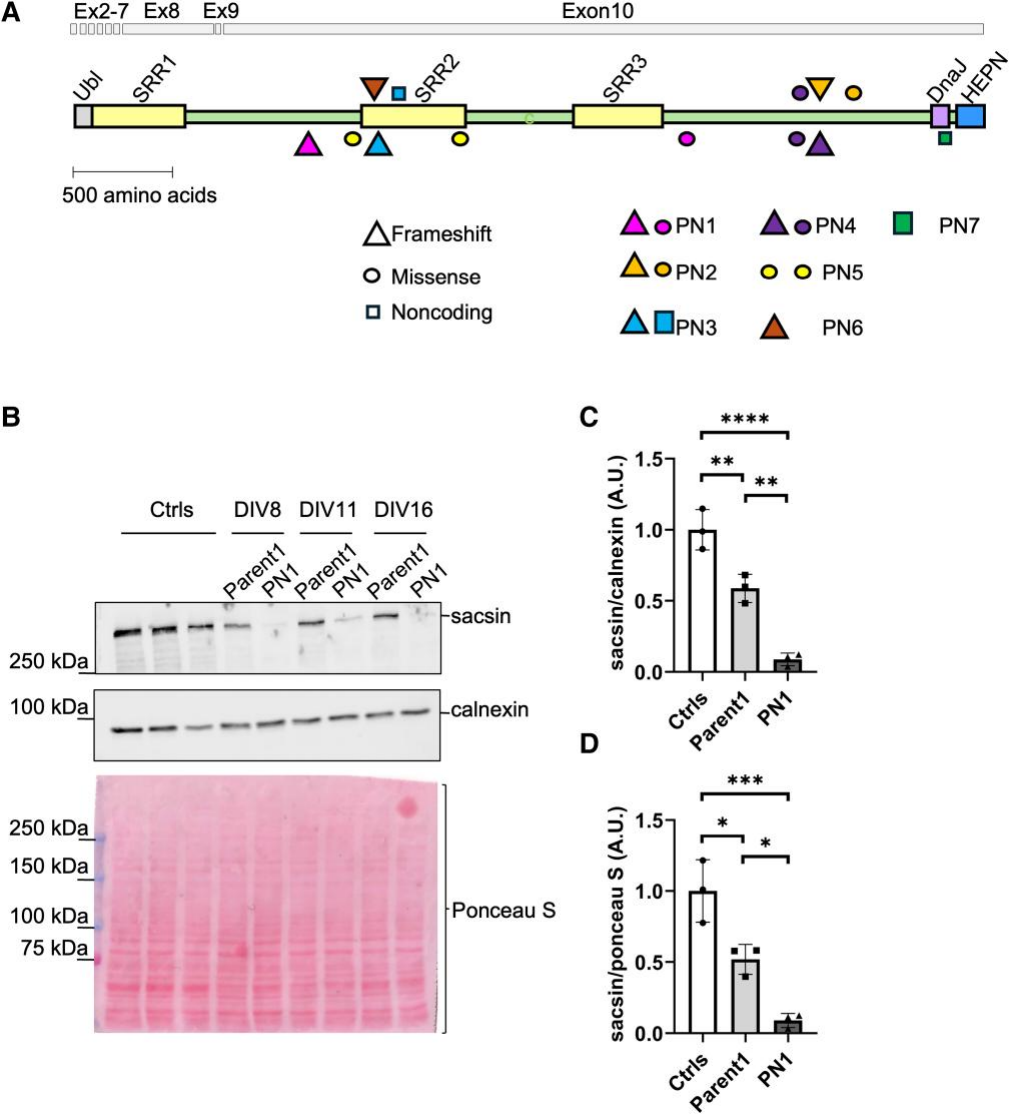
**Sacsin levels are drastically reduced in patients with ARSACS and carriers’ PBMCs carrying pathogenic missense variant.** (**A**) Schematics of sacsin protein structure; the relative positions of sacsin domains (Ubl, SRR1-3, DnaJ and HEPN) and pathogenic variants reported in this study are shown; colour code represents the different patients; shapes represent the different types of pathogenic variants (triangle = frameshift, circle = missense, square = noncoding). Corresponding positions of coding exons (abbreviated as Ex) is also shown above the protein. (**B**) Western blot showing sacsin levels in PBMCs from controls, Parent1 and PN1; three biological replicates were collected at DIV8, DIV11 and DIV16. Calnexin and Ponceau S were used as loading controls. Quantification, relative to (**B**), of sacsin levels normalized to calnexin (**C**) and Ponceau S staining (**D**). Data are presented as mean ± SD; *P < 0.05; **P < 0.01; ***P < 0.001; ****P < 0.0001 (one-way ANOVA with Tukey’s correction for multiple comparison).

Our aim was to validate the reduction in sacsin protein levels in blood samples as a diagnostic tool for *SACS* pathogenic variants. To this end, firstly we optimized a protocol for the detection of sacsin in PBMCs and secondly we provided evidence that sacsin carrying missense pathogenic variants is cotranslationally degraded in the very same blood cells, in both patients with ARSACS and healthy carriers. All the patients analysed in this study were re-evaluated by neurologists of each center in 2023–24, and the clinical scores are reported in Table 1. The *SACS* pathogenic variants analysed are reported in Fig. 1A.

We started analysing a novel compound heterozygote ARSACS patient (PN1) that we identified by NGS (Table 1) carrying a missense pathogenic variant on one allele and a frameshift on the other allele (p.Pro3095Leu; p.Leu1171Glyfs*8). Sacsin protein level was measured by western blot in PBMCs isolated from fresh venous blood and maintained in culture for diverse DIVs: DIV8, DIV11 and DIV16 (Fig. 1B). There was around a 50% reduction in the abundance of sacsin protein in Parent1 (carrier of the missense p.Pro3095Leu), as the sacsin level mean in Parent1 with respect to controls was: 59±10% SD when normalized to calnexin (*P* < 0.01) (Fig. 1C) and 52±11% SD when normalized to total Ponceau S staining (*P* < 0.05) (Fig. 1D). These data: (i) confirm that sacsin encoded by the allele carrying the missense pathogenic variant undergoes cotranslational degradation also in the PBMCs of the healthy carrier and (ii) corroborate the high reproducibility and reliability of our quantitative protocol. Consistently, sacsin levels in PBMCs of PN1 were reduced by 91±4% SD when normalized to calnexin (*P* < 0.0001) and 91±5% SD when normalized to Ponceau S (*P* < 0.001) (Fig. 1C and D). Since sacsin levels did not change between the different DIVs, we conducted subsequent experiments between DIV6 and DIV8.

**Figure 2:**
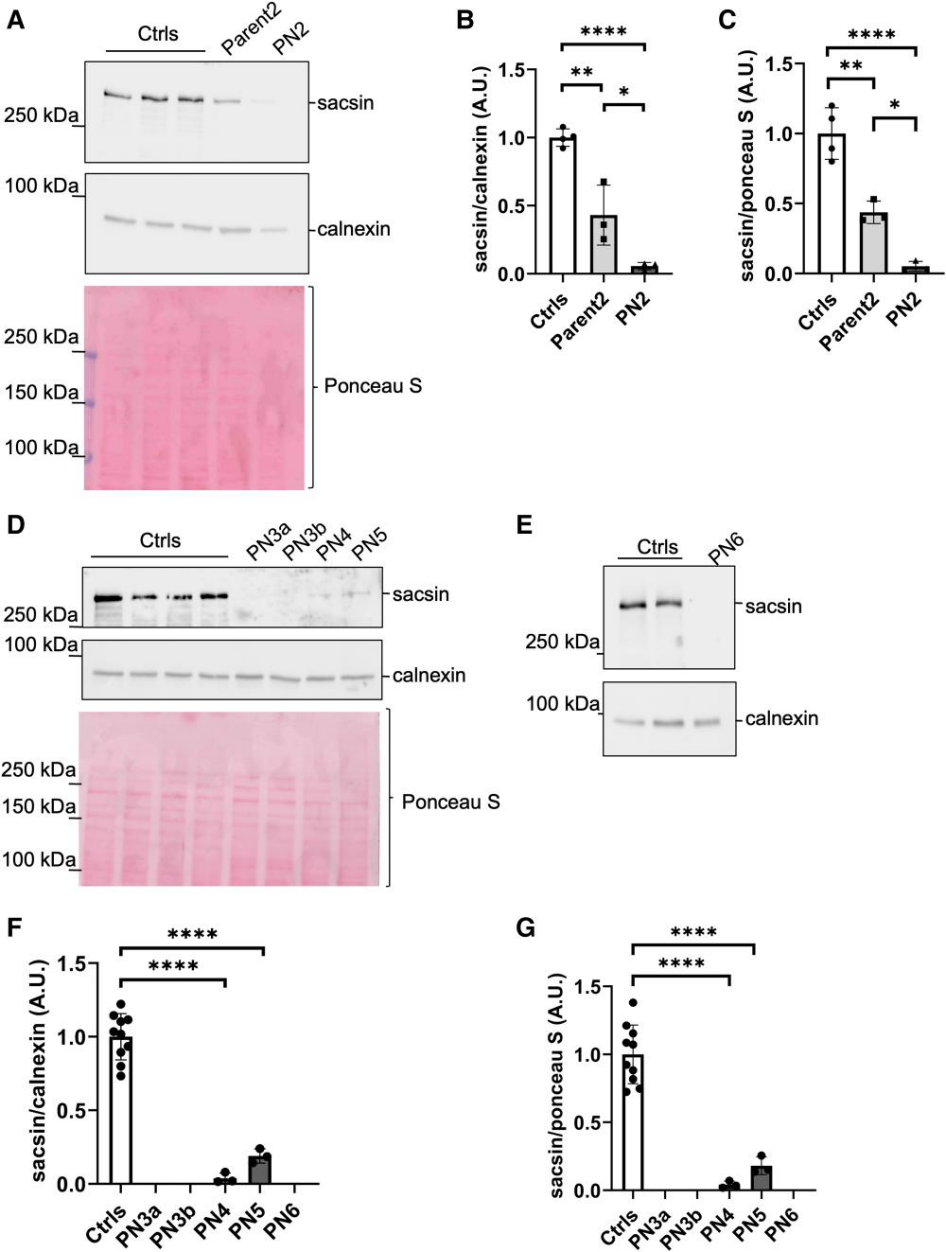
**Sacsin levels are drastically reduced in PBMCs of patients with ARSACS regardless of the type of genetic pathogenic variant.** (**A**) Representative western blot showing sacsin levels in PBMCs from controls, Parent2 and PN2; calnexin was used as a loading marker. The upper part of Ponceau S staining is also shown as loading control (see also Supplementary material for uncropped images). Quantification of sacsin levels normalized to calnexin (**B**) and total Ponceau S staining (**C**). Data are presented as mean ± SD; *N* = 3 independent western blots; *P < 0.05; **P < 0.01; ****P < 0.0001 (one-way ANOVA with Tukey’s correction for multiple comparison). (**D** and **E**) Representative western blots showing sacsin levels in PBMCs from a panel of patients with ARSACS and controls. Calnexin was used as a loading marker. The upper part of Ponceau S staining is also shown as loading control (see also Supplementary material for uncropped images). Quantification of sacsin levels normalized to calnexin (**F**) and total Ponceau S (**G**) in controls, PN3a and b, PN4, PN5 and PN6. *N* = 3 independent western blots. Data are presented as mean ± SD; ****P < 0.0001 (one-way ANOVA with Tukey’s correction for multiple comparison).

To further enforce this evidence, we then analysed PBMCs from another compound heterozygous Patient (PN2) carrying a missense pathogenic variant on one allele and a frameshift on the other allele (p.Asp3926Gly; p.Ile3755fs*8)^9^ (Table 1). Western blot on PBMCs again showed that sacsin protein level normalized to calnexin was reduced (Fig. 2A) by 57±22% SD in Parent2 harbouring the monoallelic missense pathogenic variant (p.Asp3926Gly) compared to controls (*P* < 0.01) and by 94±3% SD in PN2 compared to controls (*P* < 0.0001) (Fig. 2B). Also, normalizing to total Ponceau S re-confirmed 56±8% SD reduction in Parent2 (*P* < 0.01) and 95±4% SD reduction in PN2 (*P* < 0.0001) (Fig. 2C).

We then analysed PBMCs from other patients with ARSACS that included: two compound heterozygote siblings with two truncating variants (PN3a and PN3b) (p.Ser1531fs*9; Arg1645X)^7^ who are expected to present the complete absence of sacsin protein; one compound heterozygote with two missense *in cis* and a truncating variant (PN4) (p.Arg3636Gln, p.Pro3652Thr; p.Leu3745fs*1),^10^ one compound heterozygote with two different missense (PN5) (p.Asp1402Val; p.Trp1946Arg)^11^ and one homozygote with a truncating variant (p.Ser1531fs*9) (PN6).^12^ All patients displayed a complete or striking reduction in sacsin levels in PBMCs, regardless of the type of pathogenic variant (Fig. 2D and E). Sacsin level was not quantifiable at all in PN3a–b and in PN6 as expected, while it was reduced by 96±4% SD in PN4 (*P* < 0.0001) and by 82±7% SD in PN5 (*P* < 0.0001), normalizing to both calnexin (Fig. 2F) and Ponceau S (Fig. 2G). We also treated controls and PN4 PBMCs with the proteasome inhibitor MG-132 at 1 μM for 3 h. As we previously showed in the fibroblasts of the same patient,^7^ the levels of full-length sacsin were identical with or without MG-132 (Supplementary Fig. 1A), indicating that post-translational degradation is not the mechanism accounting for mutant sacsin reduction in PBMCs of patients with ARSACS.

**Figure 3:**
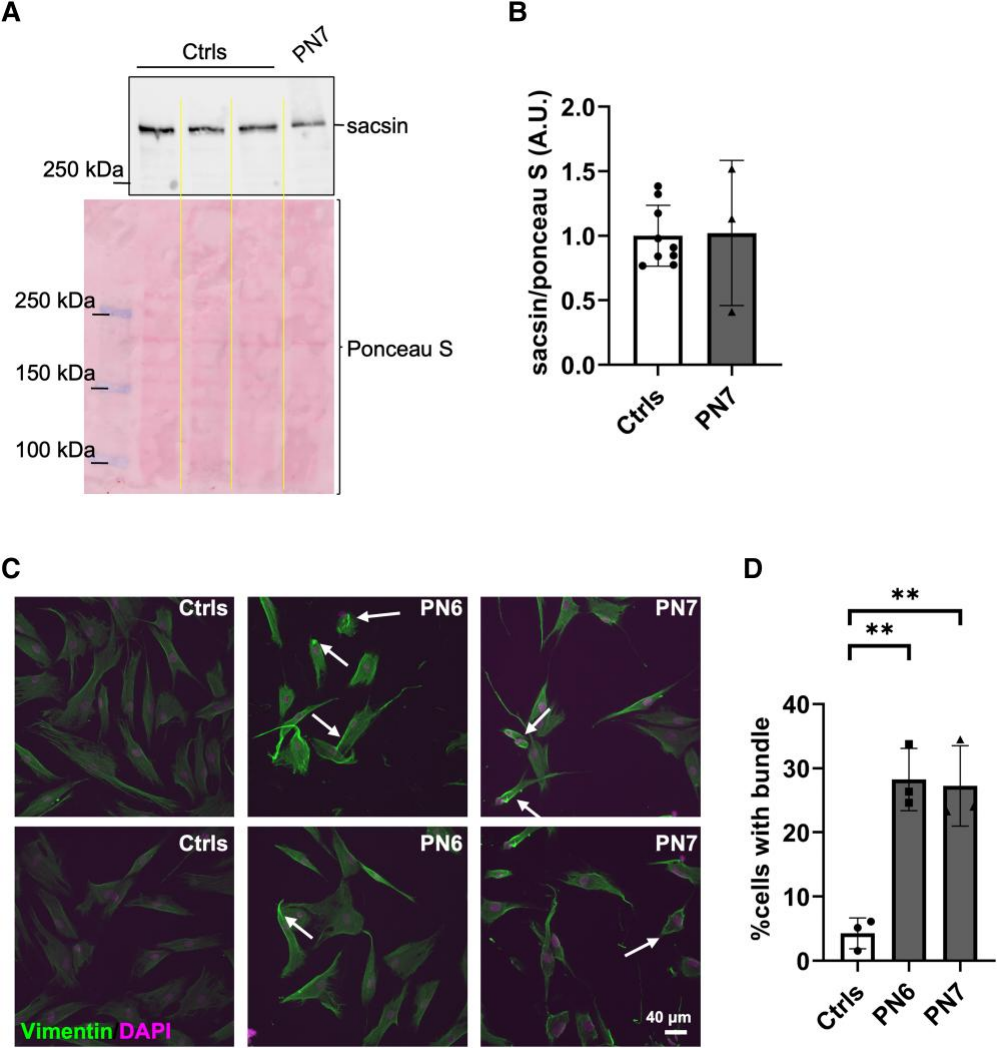
**Sacsin levels are conserved in a patient with unusual ARSACS carrying a homozygous truncating pathogenic variant near the 3′ end of the *SACS* gene.** (**A**) Representative western blot showing sacsin levels in controls and PN7. SDS–PAGE was performed with polyacrylamide gel at 5%; however, this was not resolute enough to observe the shift at this high molecular weight (from 520 to 500 kDa). Ponceau S staining is shown as loading control. (**B**) Quantification of sacsin levels normalized to Ponceau S staining signal in PN7 PBMCs compared to controls; data are presented as mean ± SD; *N* = 3 independent western blots; sacsin level in PN7 is not significantly altered with respect to controls (unpaired *t*-test). (**C**) Representative immunofluorescence images at 20× of primary fibroblasts from controls, PN6 and PN7, stained with anti-vimentin antibody (green) and DAPI (4′,6-diamidino-2-phenylindole, dihydrochloride) (magenta). White arrows indicate examples of cells in which vimentin is bundled or displaced to the periphery. Scale bar = 40μm (**D**) Quantification of the percentage of cells presenting vimentin remodelling as in (**C**), in PN6 and PN7 compared to controls; *N* = 3 independent experiments, at least 100 cells were acquired for each experiment. **P < 0.01 (one-way ANOVA with Tukey’s correction for multiple comparisons).

Finally, we also analysed sacsin protein levels from an unusual case that was previously described, in which the Patient (PN7) harbours a nonsense variant in homozygosity due to paternal uniparental isodisomy (c.13132C > T, p.Arg4378X).^13^ This truncating variant is noteworthy as it occurs near the 3′ end of *SACS* gene, leaving out the last part of the DnaJ domain and the HEPN domain in sacsin protein (see Fig. 1A and Supplementary Fig. 1B). Sacsin was detected in PBMCs from PN7 at levels comparable to those of the controls (Fig. 3A), without significant changes (Fig. 3B), suggesting that very terminal truncating variants can escape mRNA instability and degradation and can produce a nonfunctional protein. The expected molecular weight shift of 22 kDa due to the truncation was not observable by performing SDS–PAGE (even with a 5% gel), suggesting that higher-resolution methodologies are necessary to observe the shift between 520 and 500 kDa. The p.Arg4378X variant has been already reported *in trans* with another pathogenic variant in the *SACS* gene (c.1178_1181delAT; p.Leu393Cysfs*17) in a patient with ARSACS.^14^ We further validated the pathogenicity of this variant by assaying vimentin remodelling by immunofluorescence in primary fibroblasts from PN7 and comparing to PN6 and healthy controls. Intermediate filament remodelling is a hallmark of ARSACS pathogenesis, with abnormal bundles of vimentin in fibroblasts of patient with ARSACS^15^ and of neurofilaments in brain autopsies of patients with ARSACS and in the *Sacs^−/−^* mouse.^8,16^ While in the controls vimentin appeared evenly distributed in the cells, in PN6 and PN7 we observed abnormal reorganization of vimentin filaments, which appear to form bundles or to be more densely packed at the periphery and were indistinguishable between the two patients (Fig. 3C and D). Considering that PN6 has no residual sacsin protein, this experiment suggests that the homozygous p.Arg4378X variant in PN7 impairs sacsin function.

## Discussion

Classifying missense variants into pathogenic or benign remains a major challenge in the context of personalized medicine. Although improvements have been made in prediction of pathogenicity via computational algorithms,^17^ only appropriate functional assessments can give definite proof. Here, we show that depletion of sacsin due to cotranslational degradation in the presence of pathogenic missense variants can be exploited for ARSACS diagnosis. We previously formally demonstrated this mechanism in ARSACS fibroblasts,^7^ and here, we precisely quantified sacsin protein reduction in PBMCs from patients with ARSACS and healthy carriers that had one or two pathogenic missense variants. Indeed, sacsin protein was reduced by more than 80% in these patients with ARSACS compared to controls, as well as in patients with ARSACS with truncating variants that eliminate sacsin production. These data support our hypothesis that cotranslational degradation is likely a universal mechanism for pathogenic *SACS* missense variants.

We also previously demonstrated that sacsin depletion was due to mRNA degradation and instability in the case of truncating variants.^7^ Here, we report an unusual case (PN7)^13^ in which the truncating p.Arg4378X variant escapes this mechanism. This is likely due to the fact that this variant localizes at the very 3′ end of the *SACS* gene, while in our previous report the truncating variants were located further upstream.^7^ Since the p.Arg4378X is expected to result in the loss of part of the DnaJ domain and of the HEPN domain, this finding raises the intriguing hypothesis that both these domains are central in ARSACS pathogenesis. At the best of our knowledge, this is the first time that such a hypothesis is corroborated by functional data in patients with ARSACS. Because the HEPN domain binds nucleotides, it has been proposed that it is essential for the function of other sacsin domains (e.g. the Hsp40-like DnaJ domain that binds Hcs70 *in vitro* and the Hsp90-like SRR domains) by increasing local nucleotide concentration.^5^ Interestingly, in eukaryotes, the HEPN domain is almost exclusively present in sacsin-like proteins always C-terminal to the DnaJ domain,^3^ suggesting that the combination of both domains is essential for sacsin function.

Overall, these data show that assessment of sacsin protein levels could be implemented in diagnostics for ARSACS. The quantitative reduction in sacsin levels in the case of missense VUS allows defining them as pathogenic variants. Compared to truncating variants, the real effect of pathogenic missense variants on sacsin protein can be much harder or impossible to predict bioinformatically. This is even more relevant in ARSACS given that missense changes are the great majority of ARSACS-causing variants.^6^

The use of blood samples, and particularly PBMCs, for biomarker and diagnostic research has raised significant attention in recent years, as blood tissue is easily accessible and allows minimally invasive and cost-effective analyses that could be set up in diagnostic laboratories. The protocol we developed allows for the culturing, repeated expansion and freezing of PBMCs and is also set up for very low amount of material, down to 10 µg of lysate. The robustness and reliability of our protocol allow for the quantitative measure of 50% sacsin level reduction in healthy carriers, even with this low amount of protein extract. Noteworthy, this approach enables the repeated use and storage of samples for later functional analysis, without necessitating to recall patients. Although we detailed the protocol in Materials and methods allowing reproducibility by other laboratories, we are aware that the overall approach could not be immediately implementable for diagnostic laboratories without specialized knowledge anyway, and this could represent a limitation.

## Conclusion

In conclusion, this study demonstrates the effectiveness of measuring sacsin protein levels as a direct and efficient ARSACS diagnostic outcome, exploiting the underlying cotranslational degradation mechanism.

## Supplementary Material

fcae243_Supplementary_Data

## Data Availability

Anonymized data not published within this article can be made available upon reasonable request from any qualified investigator.
