# Supplementary material for: Reduction of sacsin levels in peripheral blood mononuclear cells as a diagnostic tool for spastic ataxia of Charlevoix–Saguenay
Source: Brain Commun. 2024 Jul 18;6(4):fcae243. doi: 10.1093/braincomms/fcae243 (PMC11291951; doi:10.1093/braincomms/fcae243)

Supplementary Methods

Targeted NGS panel

The targeted regions were designed to include coding exons with intronic 20 bp flanking sites. The sequencing libraries were prepared from genomic DNA by using a Sure Select enrichment system (Agilent Technologies) and run on NextSeq platform according to the manufacturer's instructions (Illumina, San Diego, CA, USA). The sequenced reads were then aligned to reference target regions and variant were called with BWA enrichment application (which also include GATK for variant calling) available on BaseSpace Sequence HUB (Illumina, San Diego, CA, USA). ANNOVAR was used for annotation against the RefSeq database and the Single Nucleotide Polymorphism databases. The filtering strategy we applied led us to select only variants located in the coding regions, including the splice site, (synonymous variants were excluded), variants that exhibited a MAF <1% or were not present in variant databases including those of the 1,000 Genomes Project and Genome Aggregation database gnomAD.

Selected variants were then analyzed with different available tools to predict possible pathogenicity such as DANN, DEOGEN2, EIGEN, FATHMM-MKL, M-CAP, MVP, MutationAssessor, MutationTaster, PrimateAI, REVEL, Polyphen-2 and SIFT.

Gene list (231) of the targeted NGS panel

AARS, ABCD1, ADAR, ADD3, AFG3L2, ALDH18A1, ALS2, AMPD2, ANO10, AP4B1, AP4E1, AP4M1, AP4S1, AP5Z1, APTX, ARHGEF10, ARID1A, ARID1B, ARL6IP1, ARSI, ASAH1, ATL1, ATL3, ATM, ATN1, ATP13A2, ATP1A2, ATXN10, B4GALNT1, BICD2, BSCL2, C10ORF2, C12ORF65, C19ORF12, CABC1, CACNA1A, CACNA1G, CACNB4, CAPN1, CASK, CAV1, CCDC88C, CHCHD10, CHMP1A, CHP1, COA7, COASY, CP, CPT1C, CYP27A1, CYP2U1, CYP7B1, DARS, DARS2, DCAF17, DCTN1, DDHD1, DDHD2, DGAT2, DNM2, DNMT1, DSTYK, DYNC1H1, EEF2, EGR2, ELOVL4, ELOVL5, ENTPD1, EP300, EPT1, ERLIN1, ERLIN2, EXOSC3, FA2H, FARS2, FAT1, FAT2, FGD4, FGF14, FIG4, FLRT1, FMR1, FTL, FXN, GAN, GARS, GBA2, GBE1, GDAP1, GDAP2, GJB1, GJC2, GRID2, GRM1, GRN, GSN, HEXA, HEXB, HSPB1, HSPB3, HSPB8, HSPD1, IBA57, IFRD1, IGHMBP2, ITPR1, KANK1, KCNA1, KCNC3, KCND3, KIAA0196, KIAA0226, KIF1A, KIF1B, KIF1C, KIF26B, KIF5A, L1CAM, LITAF, LMNA, MAG, MARS, MARS2, MFN2, MME, MORC2, MPZ, MRE11A, MSTO1, MTHFR, MTPAP, MTTP, NARS2, NEFL, NEK1, NFASC, NIPA1, NOP56, NPC1, NT5C2, OPA1, OPTN, PANK2, PDYN, PEX10, PEX7, PGAP1, PHF21A, PHYH, PLA2G6, PLD3, PLP1, PLXNA2, PMM2, PMP22, PNPLA6, POLG, POLR3A, POLR3B, PRKCG, PRRT2, PYGM, RAB3GAP2, RAB7A, RARS2, REEP1, REEP2, RNF170, RTN2, SACS, SARS2, SCP2, SCYL1, SEPSECS, SETX, SH3TC2, SIGMAR1, SIL1, SLC1A3, SLC2A1, SLC33A1, SLC9A6, SOD1, SPAST, SPG11, SPG20, SPG21, SPG7, SPTAN1, SPTBN2, STUB1,

SYNE1, SYNE2, TACO1, TBCD, TBCE, TBK1, TBP, TDP1, TECPR2, TFG, TGM6, TK2, TMEM240, TRIM2, TRMT5, TRPC3, TRPV4, TSEN2, TSEN34, TSEN54, TTBK2, TTPA, TUBA4A, TUBB2A, UBA5, UBR4, USP8, VAMP1, VAPB, VCP, VPS37A, VRK1, WDR45B, WDR48, XRCC1, XRCC4, YARS, ZFR, ZFYVE26, ZFYVE27.

Proteasome inhibition with MG-132

PBMCs at DIV6 were plated at 2*10^6 cells/mL density in a complete medium with 1µM MG-132 (Merck) for 3 hours, as previously described.[1] Control cells were treated with the same volume of MG-132 solvent DMSO (Merck). After that, cells were collected and lysed for Western Blot analysis as previously described. MG-132 efficacy was checked by Ubiquitin antibody (Abcam ab134953).

Sanger sequencing

Genomic DNA (gDNA) was extracted from PN7 PBMCs at DIV0 with phenol/chloroform. PCR amplified the region containing c.13132C>T variant with the following primers designed on *SACS* genomic sequence NG_012342.1 (RefSeqGene): FW 5' AGTGACATCTGTGGTGGAGC 3'; REV 5' TGTCTTAGCCATCTGCGTGC 3'. PCR was purified (Qiagen Kit) and sent for Sanger sequencing with both primers (Eurofins Genomics).

Supplementary References

1. Longo F, Benedetti S, Zambon AA, et al. Impaired turnover of hyperfused mitochondria in severe axonal neuropathy due to a novel DRP1 mutation. *Hum Mol Genet*. Jan 15 2020;29(2):177-188. doi:10.1093/hmg/ddz211

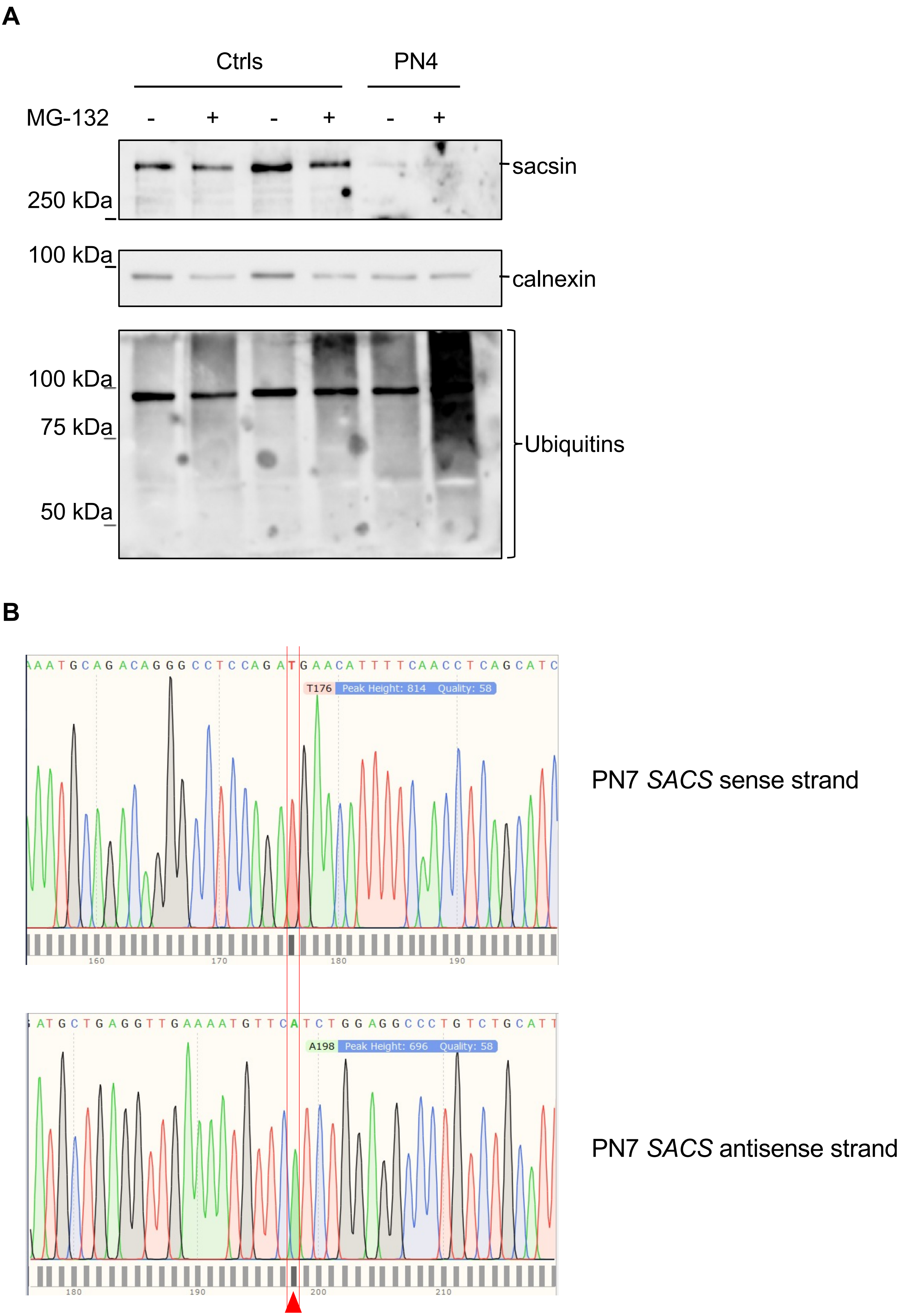

Supplementary Figure 1. (A) Representative Western Blot showing sacsin levels after 1µM MG-132 (+) or vehicle DMSO (-) treatment for three hours in PBMCs from two healthy controls and PN4. Calnexin was used as loading marker; the efficiency of MG-132 was checked with anti-ubiquitin antibody. (B) Electropherogram results of PCR Sanger sequencing for both the sense and antisense strands, using the two primers employed in the PCR amplification. The sequence data for each strand is aligned. The red lines highlight the c.13132C>T variant, which is found in homozygosis. Reference sequence from NG_012342.1 (RefSeqGene).

Full Unedited blots

Figure 1 B

Colorimetric chemiluminescence

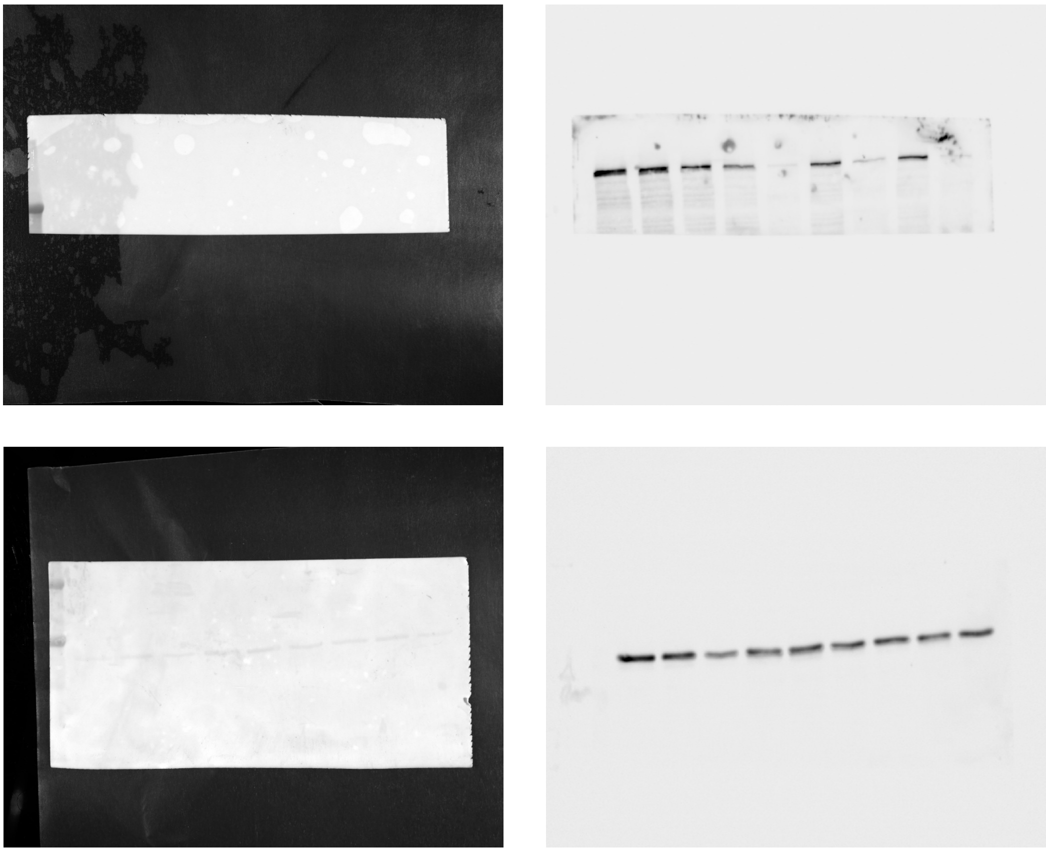

sacsin

calnexin

Ponceau staining

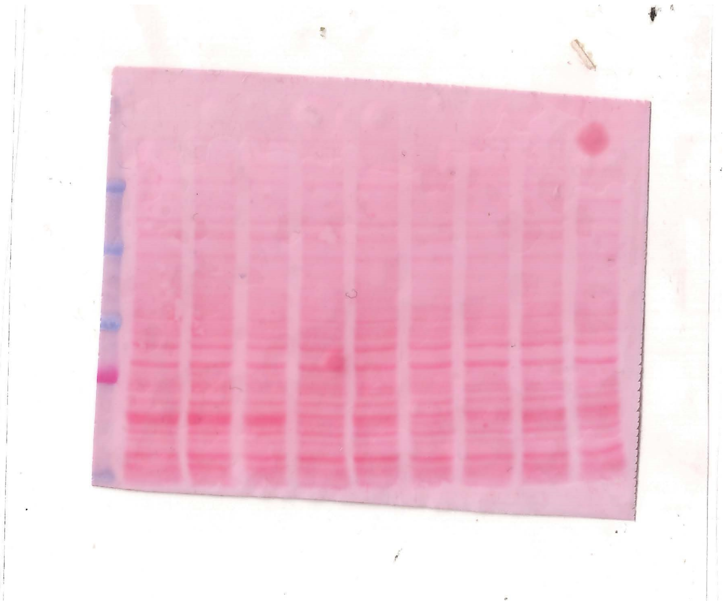

Figure 2 A

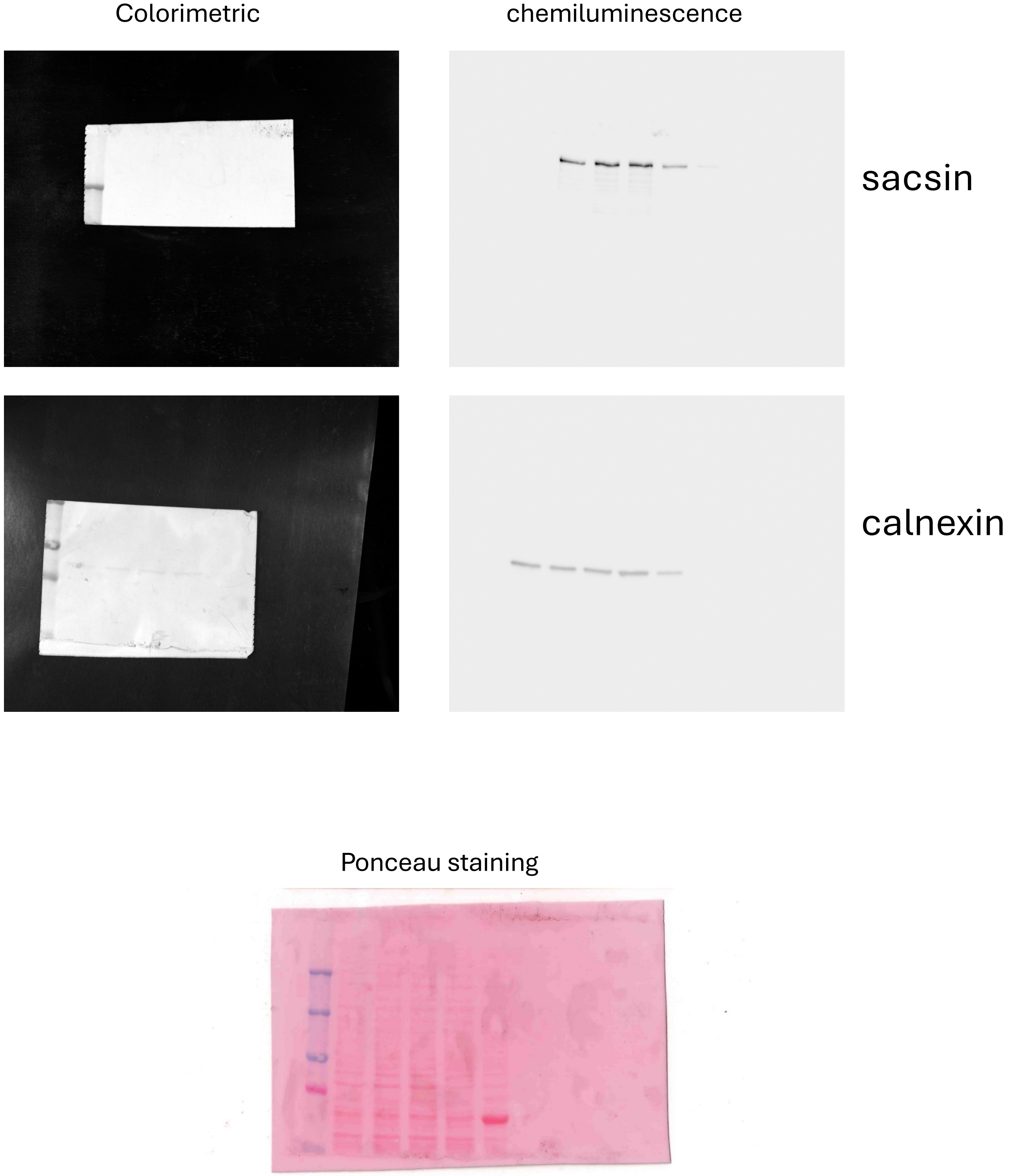
Colorimetric
chemiluminescence
sacsin
calnexin
Ponceau staining

Figure 2 D

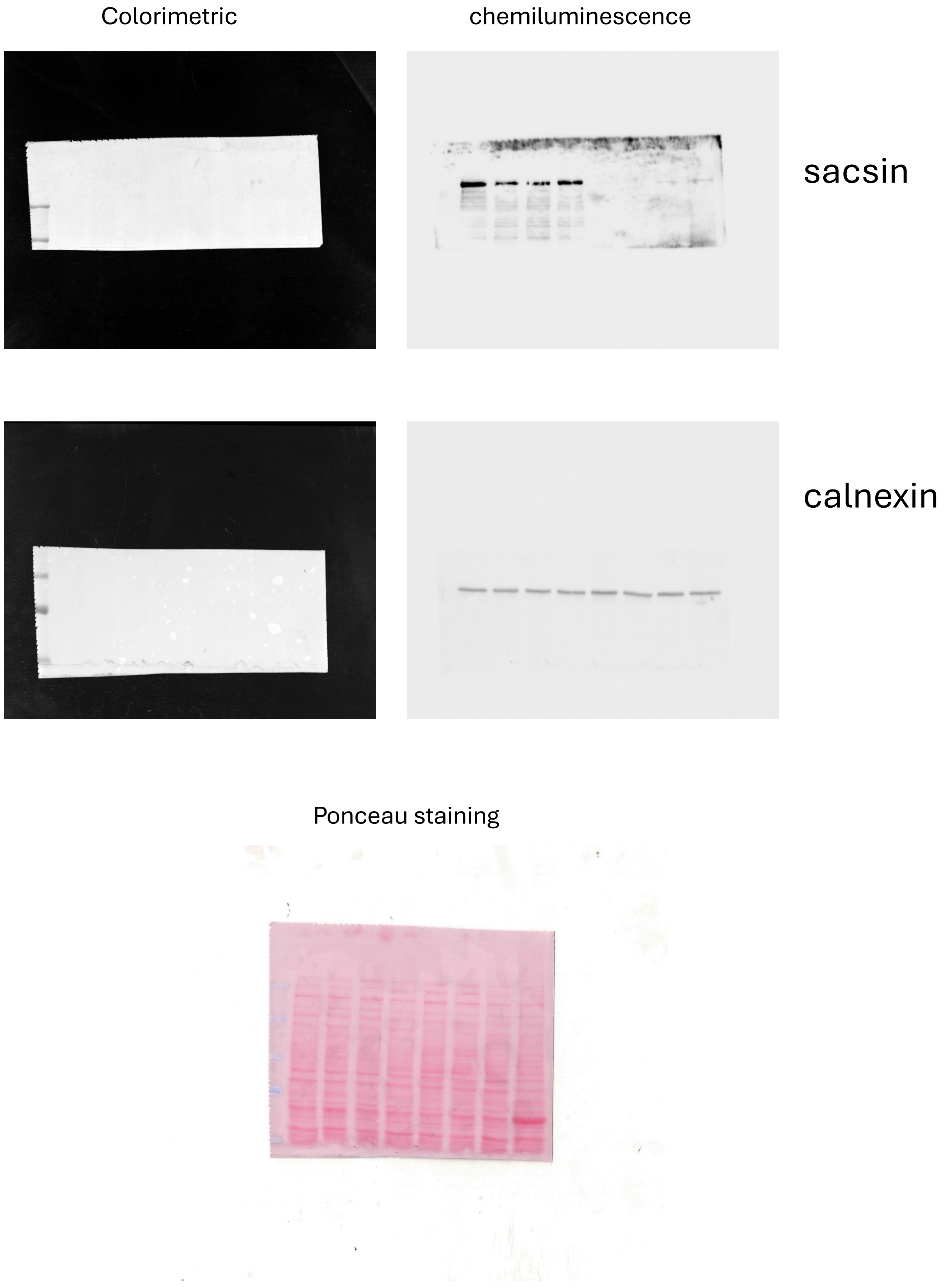
Colorimetric
chemiluminescence
sacsin
calnexin
Ponceau staining

Figure 2 E

In red square the region cropped in Fig. 2 E

Colorimetric

chemiluminescence

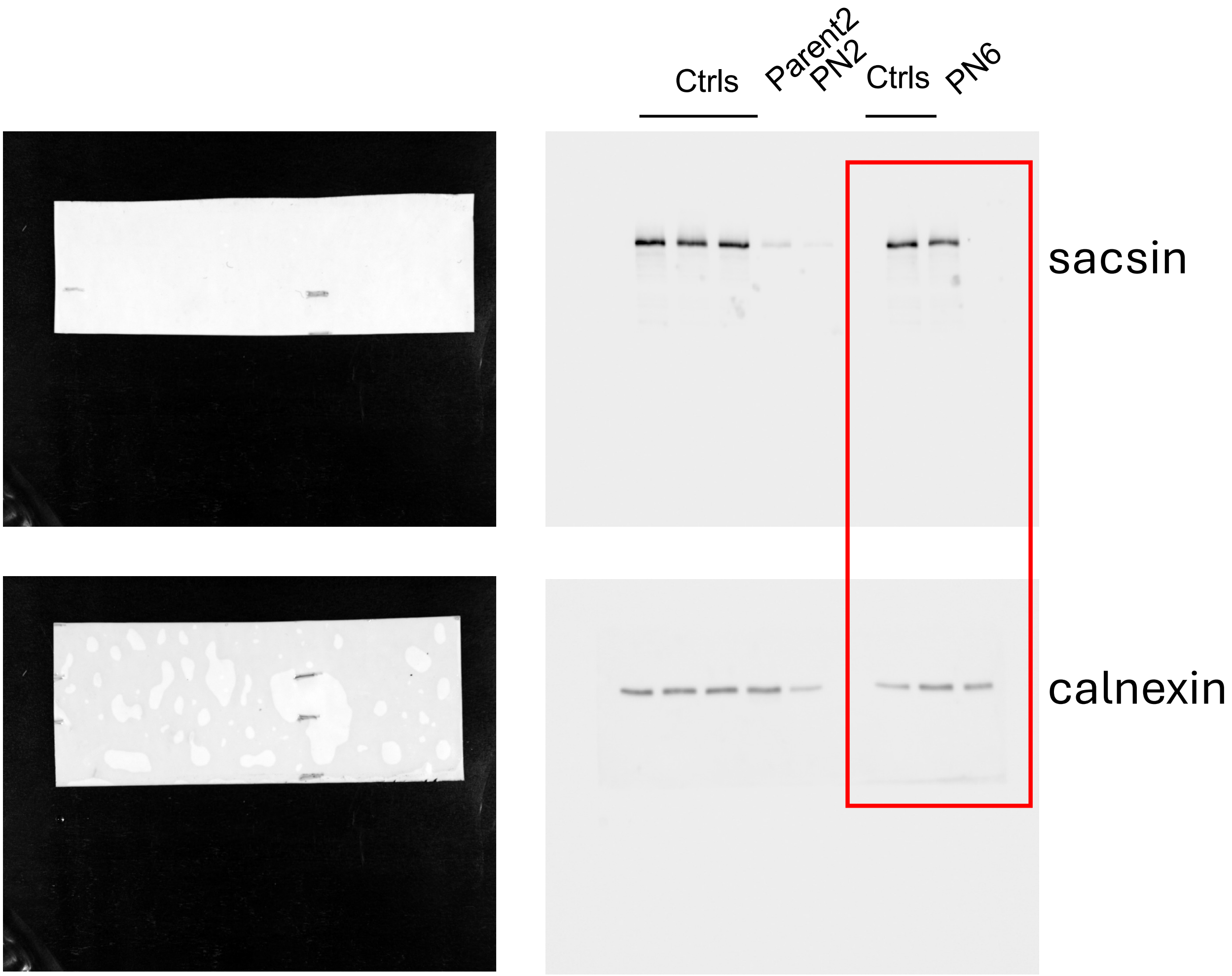

Ponceau staining

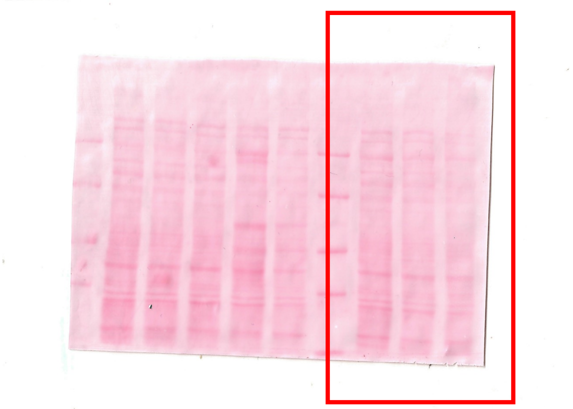

Figure 3 A

Colorimetric

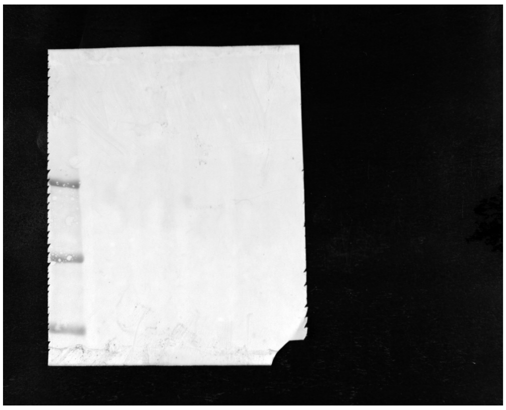

chemiluminescence

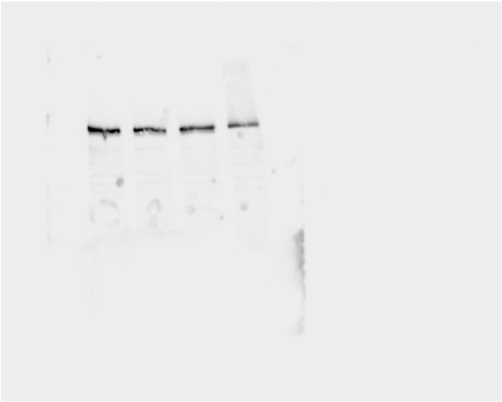

sacsin

Ponceau staining

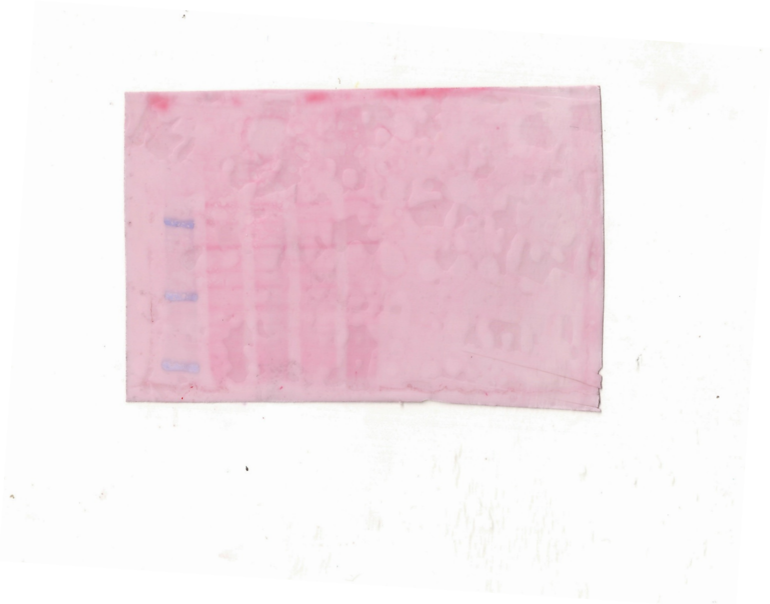

Supplementary Figure 1 A

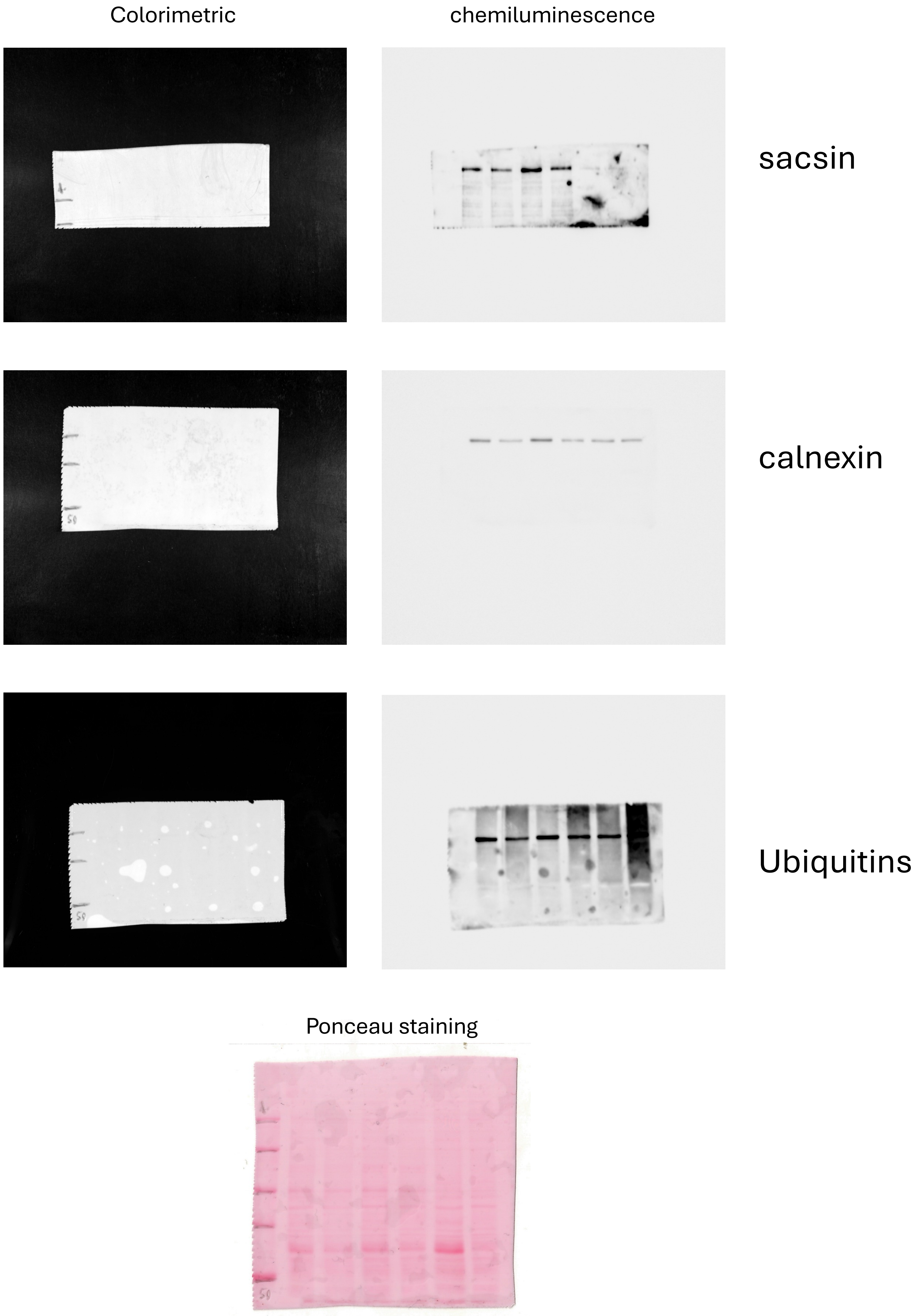

Supplement: fcae243_Supplementary_Data [file fcae243_supplementary_data.pdf]
